# Pre-exposure of *Arabidopsis* to the abiotic or biotic environmental stimuli “chilling” or “insect eggs” exhibits different transcriptomic responses to herbivory

**DOI:** 10.1038/srep28544

**Published:** 2016-06-22

**Authors:** Vivien Firtzlaff, Jana Oberländer, Sven Geiselhardt, Monika Hilker, Reinhard Kunze

**Affiliations:** 1 Institute of Biology-Applied Zoology/Animal Ecology, Freie Universität Berlin, Haderslebener Str. 9, D-12163 Berlin, Germany; 2 Institute of Biology-Applied Genetics/Dahlem Centre of Plant Sciences, Freie Universität Berlin, Albrecht-Thaer-Weg 6, D-14195 Berlin, Germany

## Abstract

Plants can retain information about environmental stress and thus, prepare themselves for impending stress. In nature, it happens that environmental stimuli like ‘cold’ and ‘insect egg deposition’ precede insect herbivory. Both these stimuli are known to elicit transcriptomic changes in *Arabidposis thaliana.* It is unknown, however, whether they affect the plant’s anti-herbivore defence and feeding-induced transcriptome when they end prior to herbivory. Here we investigated the transcriptomic response of *Arabidopsis* to feeding by *Pieris brassicae* larvae after prior exposure to cold or oviposition. The transcriptome of plants that experienced a five-day-chilling period (4 °C) was not fully reset to the pre-chilling state after deacclimation (20 °C) for one day and responded differently to herbivory than that of chilling-inexperienced plants. In contrast, when after a five-day-lasting oviposition period the eggs were removed, one day later the transcriptome and, consistently, also its response to herbivory resembled that of egg-free plants. Larval performance was unaffected by previous exposure of plants to cold and to eggs, thus indicating *P. brassicae* tolerance to cold-mediated plant transcriptomic changes. Our results show strong differences in the persistence of the plant’s transcriptomic state after removal of different environmental cues, and consequently differential effects on the transcriptomic response to later herbivory.

Plants face multiple abiotic and biotic stresses throughout their life. Their survival requires the ability to counteract adverse environmental conditions in a timely and cost-saving manner. Numerous plant species are known to successfully cope with transient stress by inducible multi-step responses regulated by hormone signalling pathways that are frequently interconnected^1^,^2^. Consequently, plant responses to a single stressful event or to multiple simultaneous environmental stresses can largely differ^3^.

In nature, certain stresses are often chronologically linked. The preceding stress may be a “warning” cue of upcoming future stress and prime the plant to prepare itself for improved resistance against impending environmental stress^4^,^5^,^6^. If the warning cue is not directly preceding a stress, a primable plant needs to retain information about the warning. Though a specific warning cue is usually expected to reliably indicate what kind of future stress will occur, and after what time lag^7^, sometimes it may also prime a plant for a different, unrelated type of upcoming stress. The “cross stress memory” of this unrelated warning may result in “cross stress tolerance”^8^,^9^.

Priming of more effective plant anti-herbivore defence has been shown in many plant species and in response to a wide range of herbivorous insects. Insect larvae that feed upon a previously warned plant were shown to perform worse and to inflict less damage than larvae on plants that have not been exposed to a warning cue. Priming of a plant for improved defence against herbivory may be elicited by previous herbivory^10^,^11^, exposure to odour of damaged leaf tissue^12^,^13^,^14^, and insect egg deposition^6^,^15^,^16^,^17^,^18^. All these warning cues are closely related to herbivory, and especially insect egg deposition is considered a cue that very reliably indicates impending larval herbivory.

Numerous studies have shown that plants can respond to insect egg deposition by mobilising their defences^19^,^20^. However, in natural environments eggs deposited on plant leaves are frequently removed, e.g. by predators or heavy rainfall. So far, it is unknown whether the plant’s response to eggs ceases after removal of the eggs, since egg removal might be taken as a cue that the danger of impending herbivory is over.

Furthermore, little knowledge is available beyond phenomenological observations how preceding, herbivory-unrelated stress such as unfavourable temperature impacts on later herbivory. In spring, it is not uncommon that feeding activity by herbivorous insects is preceded by a chilling period, because insects usually need warm temperature to hatch from eggs and to start feeding. Plants are able to acclimate to chilling conditions (‘cold acclimation’)^21^,^22^,^23^ and establish an epigenetic memory of the cold period^24^. When temperatures rise in spring, the plants deacclimate, and the retrieval of information on the previous cold exposure leads to a developmental transition. The time period that a plant requires for full deacclimation depends on the plant species^25^. For example, after only one day deacclimation from chilling temperatures the *Arabidopsis thaliana* Col-0 cold-acclimated metabolome and transcriptome are largely, but not completely reset to the state of non-acclimated plants, whereas the tolerance to subsequent freezing temperatures is still elevated^26^,^27^,^28^. However, the pace of deacclimation, including the reversion of cold-induced gene transcript levels to non-acclimated levels, varies strongly between different *Arabidopsis* accessions^28^. It is not known yet if a chilling experience can prime a plant to defend more effectively against a later herbivore attack.

In the current study, we investigated how pre-exposure of *Arabidopsis thaliana* to an environmental cue that is or is not reliably indicating upcoming herbivory affects the plant’s transcriptomic response to herbivory. We asked whether a chilling experience affects the plant’s transcriptomic response to herbivory during deacclimation to warmth, and how this response compares to the capacity to retain information about the highly reliable cue ‘egg deposition’ when the eggs have been removed prior to herbivory.

We chose *A. thaliana* and the butterfly *Pieris brassicae* as a plant–insect model system since *A. thaliana* shows inducible defence reactions against *Pieris* eggs and feeding larvae^29^,^30^,^31^,^32^ and altered transcript patterns after *Pieris* egg deposition^33^,^34^, *Pieris* larval feeding damage^35^,^36^ or exposure to cold^27^,^28^. We analysed the plant’s transcriptome after several days of chilling or egg deposition as primary stimuli and after herbivory as secondary stimulus. Since the incubation period of *P. brassicae* eggs takes about five days until larval hatching, we exposed the plants to both chilling and eggs for five days. We decided to use a short lag time of one day between primary and secondary stimuli because effects of egg deposition on the plant’s transcriptome were not expected to persist for a very long time after egg removal. This expectation is based on the assumption that maintenance of egg-induced transcriptional changes in the absence of eggs might be costly and not beneficial, because the plants are no longer threatened by imminent larval herbivory after egg removal. We chose a mild (4 °C) chilling temperature, because this induces transcript level changes in the majority of regulated genes by a magnitude that is similar to the one induced by oviposition^37^. In addition to the transcriptome analyses, we studied the performance of larvae feeding on plants that had been exposed to chilling temperature or insect egg deposition prior to herbivory. We found that (i) the chilling-induced transcriptomic excitation declines more slowly than the egg-induced excitation; (ii) one day after return to warmth the plant’s transcriptional response to herbivory differs strongly from that of chilling-inexperienced plants; (iii) in contrast, one day after removal of *P. brassicae* eggs the transcriptomic response of the plants to herbivory resembles that of plants that have never been exposed to eggs; (iv) the performance of *P. brassicae* larvae on *Arabidopsis* leaves is neither affected by preceding egg deposition nor chilling treatment.

## Results

### Transcriptional profiling of *Arabidopsis* responses to *Pieris brassicae* egg deposition and chilling

**Figure 1:**
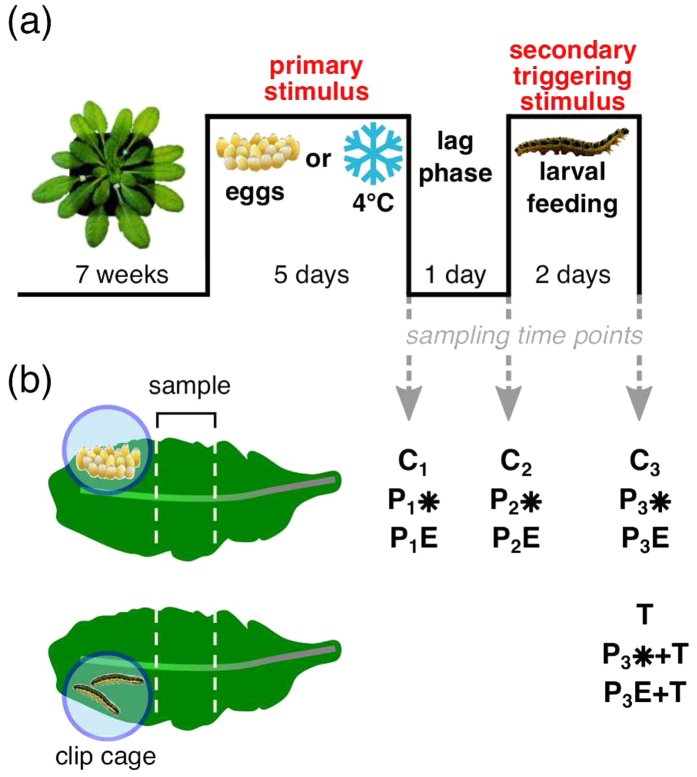
Time course of *Arabidopsis* treatment with egg deposition, chilling and/or herbivory. (**a**) Seven weeks old *Arabidopsis thaliana* plants were exposed to a primary stimulus (P) ‘*Pieris brassicae* egg deposition’ or ‘chilling at 4 °C’, respectively. After five days, the eggs were removed or plants were returned to 20 °C. After a one-day-deacclimation period a secondary stimulus ‘feeding by *P. brassicae* larvae’ was applied for two days, here referred to as secondary triggering stimulus (T). Leaf tissue (see panel **b**) was harvested at three time points: (i) after a 5-day-treatment period with the primary stimulus ‘egg-deposition’ (P_1_E) or ‘chilling’ (P_1_*) and from untreated control plants (C_1_); (ii) one day after removal of eggs from ‘egg-deposition’-treated plants (P_2_E), after one day of deacclimation of ‘chilling’-treated (P_2_*), and of untreated control plants (C_2_) of the same age; (iii) after two days with/without the secondary triggering stimulus ‘larval feeding’ of ‘egg-deposition’-treated (P_3_E + T/P_3_E), ‘chilling’-treated (P_3_* + T/P_3_*), and untreated control plants (T/C_3_). (**b**) A 1 cm wide leaf strip proximal to the ‘egg deposition’ and/or ‘larval feeding’ treated area was harvested for transcriptome analysis. From ‘chilling’-treated and untreated plants, a corresponding leaf area was harvested. The circle around the larvae indicates the clip cage trapping the larvae.

**Figure 2:**
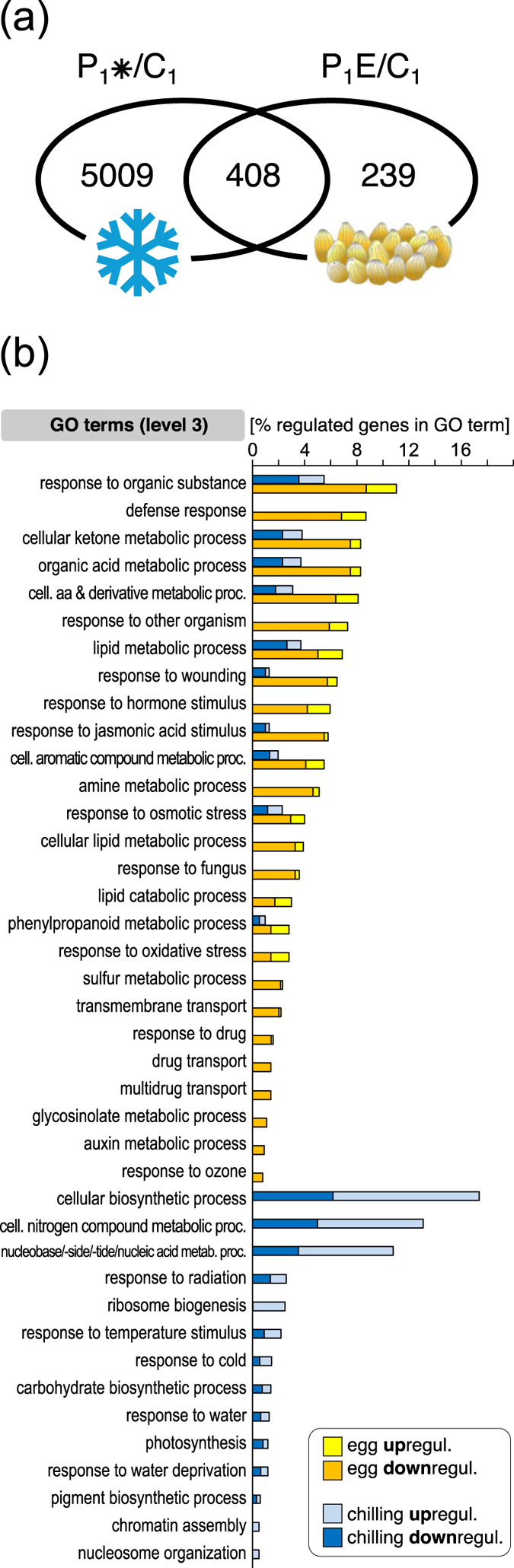
Transcriptome changes induced by chilling or egg deposition after five days. (**a**) Venn diagram illustrating the number of individual and shared genes that show ≥2-fold expression changes in ‘chilling’-treated (P_1_*/C_1_; five days at 4 °C) and ‘egg-deposition’-treated leaves (P_1_E/C_1_; five days after egg deposition by *Pieris brassicae*). Depicted are genes with expression ratio ≥2 and *P*_*adj*_ < 0.05 (*n* = 3). (**b**) Significantly enriched GO terms in egg deposition- and chilling-responsive genes. Egg deposition-responsive (yellow columns) and chilling-responsive genes (blue columns) were mapped to the GO terms in GO-level 3. The length of the bars shows the percentage of regulated genes in the respective GO categories. The brightly coloured right section of each bar represents upregulated genes; the dark coloured left section represents downregulated genes.

We compared the transcriptional responses of *A. thaliana* leaves after five days of exposure to insect egg deposition or chilling. At this first sampling time point (Fig. 1a), 647 and 5,417 genes, respectively, were transcriptionally more than 2-fold up- or downregulated when compared to untreated controls (Supplementary Table S1), and 408 of these genes were regulated by both treatments (Fig. 2a).

To determine whether the regulated genes were significantly overrepresented in distinct biological processes, the genes regulated by the chilling and egg treatment were mapped to the Gene Ontology (GO) terms. The overall number of chilling-regulated genes was more than 8 times higher than of the oviposition-affected genes. Nevertheless, the chilling-regulated genes mapped to only 24 biological processes level-3-GO terms, while the egg-responsive genes distributed to 26 level-3-GO terms. The chilling-responsive biological processes were more enriched with upregulated genes, whereas the egg-responsive ontologies were dominated by downregulated genes (Fig. 2b). After five days cold exposure the upregulated genes were most prominently overrepresented in the ‘cellular biosynthetic process’, the ‘cellular nitrogen compound metabolic process’ and ‘nucleobase/nucleoside/nucleotide/nucleic acid metabolic process’ ontologies. The terms ‘response to temperature stimulus’ and ‘response to cold’ include known cold-induced genes such as *CBF2/DREB1C*, *COR15A* and *ZAT12*^22^,^38^,^39^,^40^. The egg deposition caused regulation of genes in biological processes related to hormones such as salicylic acid (e.g. *BAP1*, *HSPRO2*), phenylpropanoid metabolism (e.g. *At2g21100, UGT72E2*), defence response and responses to other organisms (e.g. *YLS9, PME17, GLIP1*) and oxidative stress (e.g. *At1g13340, At2g44240*) (Fig. 2b; Supplementary Table S2). Interestingly, both the chilling and egg-treated plants showed regulation of genes in 10 common biological processes. These include stress related categories like phenylpropanoid metabolic processes, wounding and response to jasmonic acid stimulus. A list of all significantly enriched gene ontologies of biological processes in levels 1 to 5 is provided in Supplementary Table S2.

### Egg deposition- and chilling-induced transcriptome states wear off with different pace after stimulus removal

The persistence of the transcriptomic responses to either chilling or egg deposition was examined after termination of exposure to the primary stimulus by removing the egg clusters or returning the plants to 20 °C, respectively. The plants were left untreated for one day (second sampling time point, Fig. 1a; P_2_E and P_2_*) or for three days (third sampling time point, Fig. 1a; P_3_E and P_3_*).

**Figure 3:**
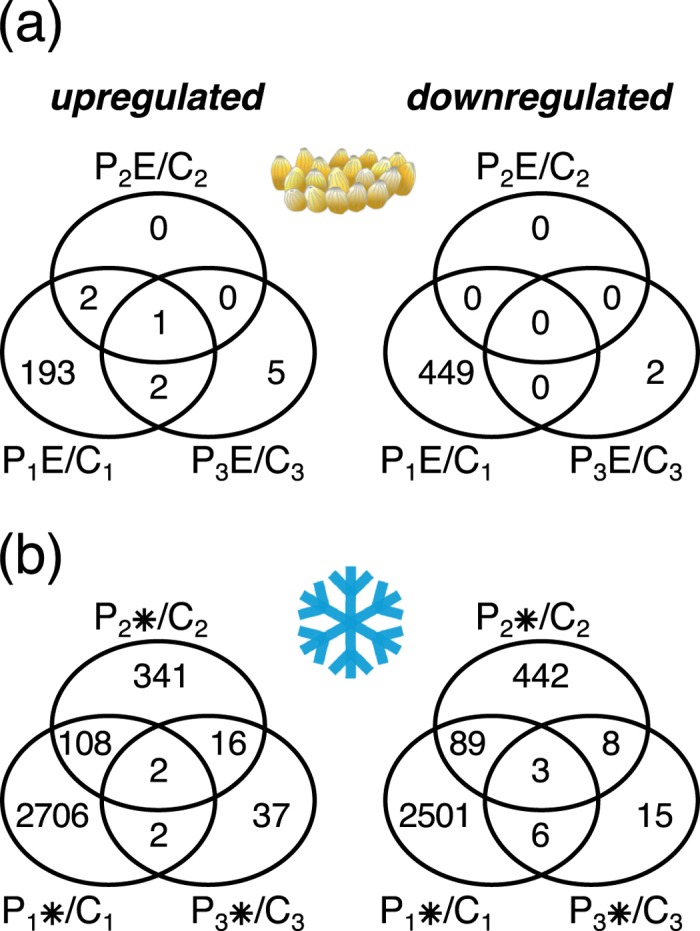
Time course of transcriptome changes in oviposited or chilled *Arabidopsis* leaves. (**a**) Upregulated (left panel) and downregulated (right panel) genes in leaf tissue next to the egg cluster five days after egg deposition (P_1_E/C_1_), one day after removal of the eggs (P_2_E/C_2_) and three days removal of the eggs (P_3_E/C_3_). (**b**) Upregulated (left panel) and downregulated (right panel) genes in leaf tissue of plants exposed to 4 °C for five days (P_1_*/C_1_), one day after returning the plants to 20 °C (P_2_*/C_2_) and three days after returning the plants to 20 °C (P_3_*/C_3_). Depicted are genes with expression ratios ≥2 and *P*_*adj*_ < 0.05 (*n* = 3; except for C_3_ with *n* = 4).

Transcription analysis after the “no treatment” phases revealed that the oviposition-induced gene expression changes relative to untreated control plants had almost completely vanished within only one day after egg- removal (Fig. 3a). Three days after egg-removal, seven weakly regulated genes appeared exclusively in previously oviposited plants (Fig. 3a, Supplementary Table S1). Of the seven genes, the five approximately 2-fold upregulated genes encode receptor-like proteins (RPP27 and RLP21), cytochrome P450 family proteins (At4g15340 and At5g38970) and a putative MATE efflux carrier (At2g04090), and thus may be related to stress responses. The two weakly downregulated genes are targets of the cytokinin- and ABA-signalling network, respectively, and may also be related to stress responses. RALF30 (At4g13075) is controlled by the type-B response regulators ARR1/10/12^41^ and the ABA-responsive gene At5g23350 encodes a GRAM-domain containing protein involved in drought-stress protection^42^. Overall, although an egg cluster on a leaf induced a strong transcriptional reaction near the deposition site, this response was only maintained as long as the egg cluster was present.

The chilling-induced expression changes had also largely declined to 1,009 regulated genes after one day deacclimation at 20 °C (Fig. 3b, P_2_*/C_2_) and to only 89 genes after three days (Fig. 3b, P_3_*/C_3_). Strikingly, of the 1,009 genes regulated after one day recovery, 357 and 450 genes were deacclimation-specifically up- and downregulated, respectively, i.e. these genes did not show a direct response to cold treatment. After three days, the number of deacclimation-specifically regulated genes had declined to 53 up- and 23 downregulated genes. Of these, 37 and 15 genes were up- and downregulated, respectively, exclusively in the P_3_* leaves three days after the return to 20 °C, but not after one day. This indicates that during deacclimation the *Arabidopsis* leaf transcriptome undergoes a specific reprogramming that involves numerous uniquely regulated genes.

For validation of the microarray results, we selected 12 genes with egg- or chilling-responsive transcript profiles, several of which had previously been reported to be egg- or chilling-induced^22^,^33^,^38^,^39^,^40^,^43^. In all cases the qRT-PCR results confirmed the microarray data (Supplementary Table S3, rows 5–16).

### Larval performance is neither affected on chilling-treated leaves nor on leaves from which eggs have been removed

Neonate larvae were placed on the leaves from which eggs had been removed one day before. After a two-day-feeding period, they gained as much weight and consumed about the same leaf area as larvae that fed on untreated leaves. The differences were not significant (Table 1). Likewise, neither did a prior chilling treatment of plants significantly affect the weight of larvae nor the extent of leaf damage inflicted by these larvae (Table 1).

### Preceding egg deposition or chilling treatment differently reprogrammes the herbivore feeding-induced *Arabidopsis* leaf transcriptome

We analysed transcriptomes of leaf tissue that had been exposed to herbivory for two days. Prior to herbivory, these plants had experienced egg deposition (P_3_E + T) or chilling (P_3_* + T) with one day lag phase between egg- or chilling-treatment and herbivory. We compared these transcriptomes (i) with those of leaves from untreated control plants (C_3_), and (ii) with those of leaves that had only been exposed to the triggering stimulus ‘herbivory’ (T), but did not experience any previous treatments.

**Figure 4:**
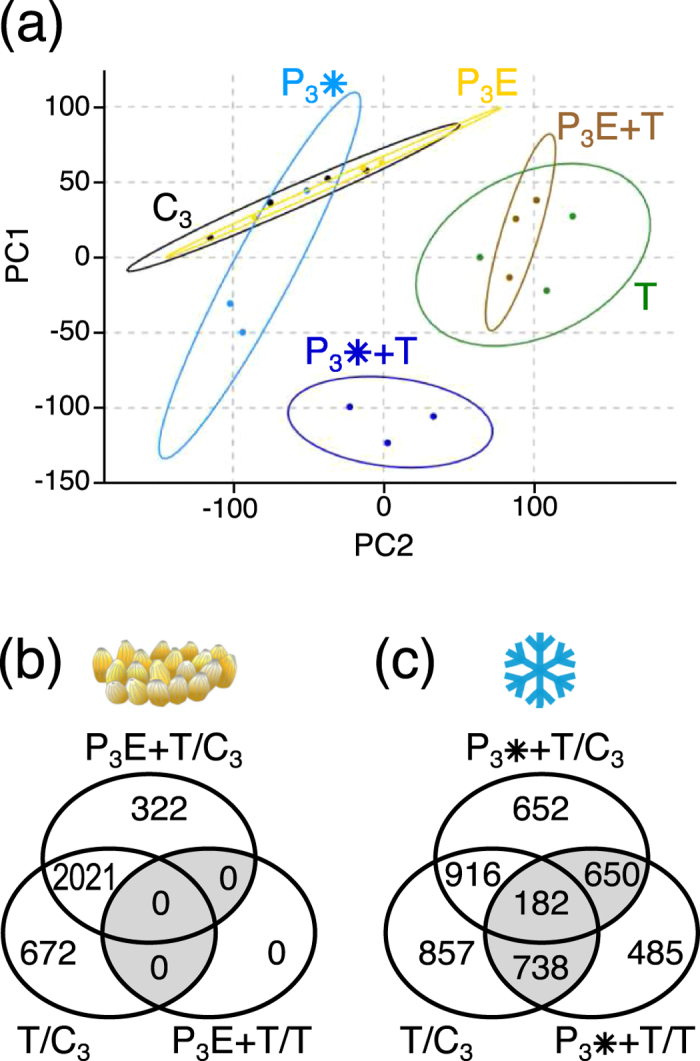
Transcriptome reconfiguration in *Arabidopsis* leaves by oviposition, chilling and herbivory. (**a**) Principle component analysis of log_2_-transformed microarray data of individual samples (biological replicates) from non-treated, chilling- or egg-treated leaves after three days recovery (C_3_, P_3_*, P_3_E) and the respective samples that were exposed to *Pieris brassicae* larval feeding (T, P_3_* + T, P_3_E + T). Depicted are the first two principal components PC1 and PC2 which explain 29.5% and 16.6% of the variance, respectively. Ellipses represent 95% confidence intervals. (**b**) Genes regulated by larval feeding on untreated plants (T/C_3_), by larval feeding on prior egg-treated plants (P_3_E + T/C_3_) and differentially regulated genes by larval feeding on prior egg-treated plants compared to larval feeding on untreated plants (P_3_E + T/T). (**c**) Genes regulated by larval feeding on untreated plants (T/C_3_), by larval feeding on prior chilling-treated plants (P_3_* + T/C_3_) and differentially regulated genes by larval feeding on prior chilling-treated plants compared to larval feeding on untreated plants (P_3_*T/T). Depicted are genes with expression ratios ≥2 and *P*_*adj*_ < 0.05 (*n* = 3; except for C_3_ with *n* = 4).

The data sets obtained from the biological replicates of each treatment were analysed by a principle component analysis (PCA) (Fig. 4a). The scatter plots of the egg-treated (P_3_E) and chilling-treated samples (P_3_*) without herbivory (harvested three days after egg removal or at the end of chilling treatment) overlapped with those of the untreated control plants (C_3_), whereas both the previously egg-treated and the chilling-treated samples from leaves that had been exposed to herbivory were clearly separate from the control. Consistent with the lack of differentially regulated genes already one day after egg removal (P_2_E/C_2_) (Fig. 3a), the egg-treated samples (P_3_E) were almost congruent with the untreated samples (C_3_) (Fig. 4a). The scatter plot of the chilling-treated samples (P_3_*) had a slightly shifted centre (Fig. 4a), reflecting the 52 genes that were differentially regulated compared to the untreated plants (Fig. 3b). Feeding-damaged samples without pre-treatment (T) and feeding-damaged samples that had experienced prior egg deposition (P_3_E + T) were almost superimposed, but separate from the unwounded samples, indicating that larval feeding alone was responsible for most of the transcriptome changes. Hence, the previous egg deposition that had been removed one day prior to herbivory did hardly affect the induction by herbivory anymore. The feeding-damaged samples that had experienced chilling (P_3_* + T) prior to herbivory located at a distinct and distant position in the scatter-plot, suggesting that the primary chilling stimulus implemented a ‘primed’ status that prompted a largely different transcriptional response to insect feeding than in ‘non-primed’ plants.

As expected, larval feeding-induced a strong transcriptional response with 2,693 significantly regulated genes relative to the untreated, non-damaged control plants (Fig. 4b, T/C_3_). When the plants had experienced first egg deposition and later herbivory, 2,343 genes showed different expression than untreated, non-damaged control plants (P_3_E + T/C_3_). A large fraction of them (87%) was also regulated in the plants that did not carry egg clusters before, but were feeding-damaged. However, when directly comparing the gene expression levels in previously egg-treated, feeding-damaged plants with those in the egg-free, feeding-damaged ones (P_3_E + T/T), it became apparent that no genes showed significantly different expression levels between these treatments.

Markedly different from the lacking effects of egg deposition on gene expression levels after egg removal, prior chilling strongly influenced the transcriptional response to feeding damage. Previously chilled plants showed specific transcriptional responses to herbivory: Only 41% of the 2,693 feeding-responsive genes in untreated plants (T/C_3_) were also regulated in previously chilled plants (Fig. 4c, P_3_* + T/C_3_). When comparing the transcriptomic response to herbivory of previously chilled plants to the one of unchilled plants, 2,055 genes showed more than 2-fold deviating expression levels (Fig. 4c, P_3_* + T/T). Among them, 485 genes appeared to be differentially (≥2-fold) regulated exclusively in the P_3_* + T/T comparison, but they showed no differential expression (<2-fold) when comparing their levels in untreated, feeding-damaged plants with those in controls (T/C_3_) or in chilling-treated, feeding-damaged plants with controls (P_3_* + T/C_3_), or *vice versa*. The remaining 1,570 genes (Fig. 4c, shaded subsets) displayed not only significant expression differences between untreated, feeding-damaged (T) and chilling-treated, feeding-damaged plants (P_3_* + T), but also between both of these types of feeding-damaged plants and the untreated control plants (C_3_).

We reassessed the microarray-derived expression data by qRT-PCR analysis of nine genes that exhibited conspicuous transcriptional regulation after feeding damage, five of them with an attenuated response after prior chilling experience. The qRT-PCR data confirmed in all cases the microarray results (Supplementary Table S3, rows 16–24).

Among the 2,055 differently regulated genes in feeding-induced, previously chilled compared to unchilled plants (Fig. 4c and Supplementary Table S1, P_3_* + T/T), only ~5% were also regulated in chilled plants after one day deacclimation at 20 °C (Supplementary Table S1, P_2_*/C_2_). This small overlap indicates that the high number of regulated genes in feeding-induced, chilled plants is not due to the persistence of transcriptional excitation of many genes during deacclimation. This is corroborated by the fact that less than 5% of the 4,880 up- or downregulated genes after five days chilling, whose expression returned to control levels after one day deacclimation (Supplementary Table S1, P_1_*/C_1_ minus P_2_*/C_2_), were again up- or downregulated in the feeding-induced, previously chilled compared to unchilled plants (Supplementary Table S1, P_3_* + T/T).

**Figure 5:**
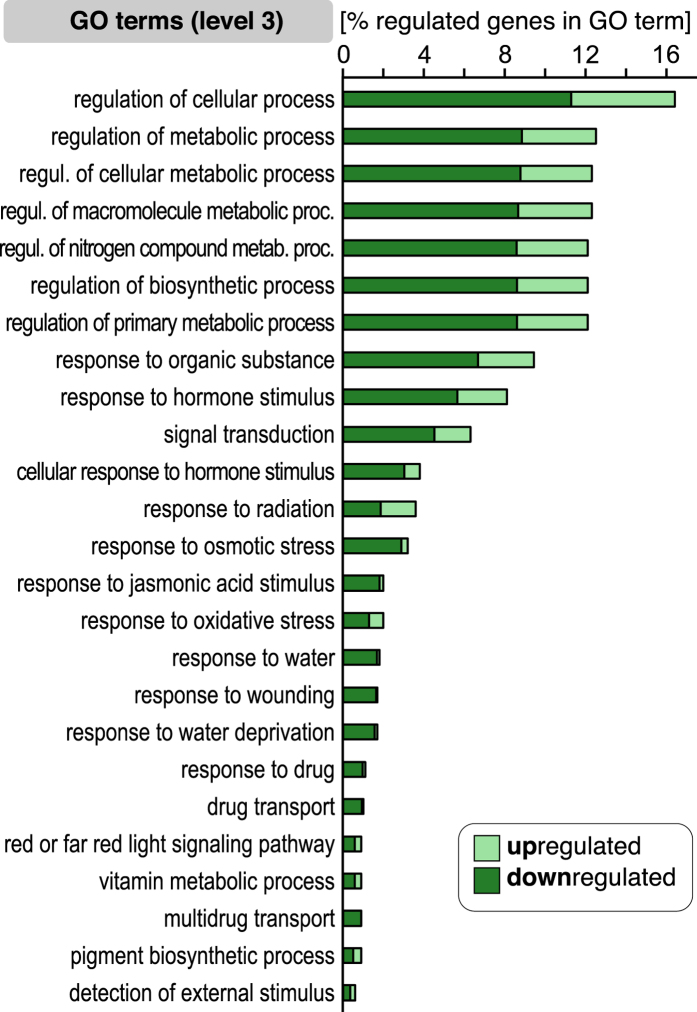
Biological process-GO terms enriched with larval feeding-responsive genes with chilling treatment-specific expression patterns. The 1,570 *Pieris brassicae* larval feeding-responsive genes with chilling treatment-specific expression patterns (shaded gene subsets in Fig. 4c) were mapped to the GO terms in GO-level 3. The length of the bars shows the percentage of regulated genes in the respective GO categories. The brightly coloured right section of each bar represents upregulated genes; the dark coloured left section represents downregulated genes.

The 1,570 differentially expressed, ‘chilling primed’, feeding-induced genes fall into 25 biological process ontologies (GO level 3; Fig. 5). Conspicuously, the majority of genes in these GO terms were downregulated in the chilling-treated, feeding-induced plants relative to the non-chilled, feeding-induced plants (Fig. 5; column “P_3_* + T/T” in Supplementary Table S1). The ontologies with the largest fractions of differentially expressed genes comprise genes involved in regulation of various metabolic or biosynthetic processes, signal transduction, and responses to phytohormones (Fig. 5). The GO categories with the highest number of regulated genes are ‘response to hormone stimulus’ and ‘response to organic substance’ with 126 and 148 genes, respectively (Supplementary Table S2). Consistent with previous studies^22^, many genes involved in the JA-pathway were downregulated in response to chilling as single stimulus (Supplementary Table S4 column P_1_*/C_1_: 106 of 126 regulated genes are downregulated). In contrast, feeding-induced predominantly upregulation of JA-pathway genes (Supplementary Table S4 column T/C_3_: 104 of 113 regulated genes are upregulated), as has also been reported in previous studies^44^. However, the plant’s response to feeding after exposure to chilling (P_3_* + T) obviously shows attenuated upregulation of JA-pathway genes when compared to feeding-induced, unchilled plants (T): 58 of 64 regulated genes in P_3_* + T samples were downregulated when compared to T samples (Supplementary Table S4 column P_3_* + T/T).

Overall, these findings revealed that the feeding-induced *A. thaliana* transcriptional response during deacclimation from a chilling stress strongly differs from that of plants grown in a constantly warm climate.

## Discussion

Our study shows that the transcriptomic recovery from a past environmental cue experienced by *A. thaliana* depends on the type of cue. While the plant retains some chilling-induced transcriptomic changes for at least a day during deacclimation to warmth, the oviposition-induced transcriptome is already reset to the egg-free state one day after removal of eggs. We further demonstrate that prior chilling affects the plant’s transcriptomic response to herbivory. In contrast, the highly reliable herbivory-indicating cue ‘egg deposition’ does no longer affect the plant’s transcriptomic response to herbivory after removal of this cue one day prior to feeding damage.

The transcriptional response of *A. thaliana* to *P. brassicae* eggs shown in this study (P_1_E/C_1_, Fig. 2) and previous ones^33^,^34^,^45^ may be considered as a defence response that is targeting the eggs. Indeed, Blenn *et al*.^30^ had shown chemical defence responses of *A. thaliana* to *P. brassicae* egg deposition. The eggs induced a change in the chemical composition of the leaf epicuticular wax layer which caused enhanced host foraging activities by egg parasitoids, thus providing indirect plant defence against the eggs with the help of parasitic insects. The egg-induced transcriptional changes of *A. thaliana* may also provide an ‘alert’ state which prepares the plant for improved anti-herbivore defence if eggs are not removed prior to herbivory. A study by Geiselhardt *et al*.^32^, who did not remove *P. brassicae* eggs from *A. thaliana* leaves prior to larval feeding, indicates that eggs are taken as ‘warning’ of impending herbivory; the *P. brassicae* larvae performed worse on previously egg-deposited than on egg-free leaves.

Interestingly, the egg-induced transcriptomic changes found in our study and in those by e.g. Bruessow *et al*.^34^ and Hilfiker *et al*.^45^ show parallels to the systemic acquired resistance (SAR) response of a plant to phytopathogens^20^. The differences between the egg-affected transcriptome (Fig. 2; P_1_E/C_1_) analysed in the current study and the one by Little *et al*.^33^ may be due to the different sampling sites (directly underneath the oviposition site^33^ *versus* adjacent to the oviposition site in our study) and to the different time points of sampling.

The *Arabidopsis* leaf transcriptome was reset within one day to the pre-oviposition status after removal of eggs from leaves (Fig. 3a; P_2_E/C_2_). Accordingly, the feeding-induced transcriptome of previously egg-deposited leaves did not differ from the feeding-induced transcriptome of egg-free leaves (P_3_E + T/T). This is also consistent with the unimpaired performance of *P. brassicae* larvae feeding on leaf tissue adjacent to the position of the former egg cluster (Table 1) and in agreement with the regular *P. brassicae* larval development on *Arabidopsis* leaves one day after egg removal described by Hilfiker *et al*.^45^.

It might be a cost-saving strategy to no longer maintain a transcriptionally excited state in response to egg deposition when the cue ‘presence of eggs’, that reliably indicates upcoming feeding damage, disappears^46^. In nature, insect eggs are frequently removed from leaves. Heavy winds, rainfall and predation may cause a loss of more than 80% of lepidopteran eggs^47^.

Chilling of the whole *Arabidopsis* plant has, not surprisingly, a much more severe impact on its metabolism and development than local egg depositions on a leaf. The extensive transcriptome reprogramming accompanying acclimation to chilling conditions that we observed after five days of exposure to chilling (Figs 2 and 3b) is in agreement with previous reports^48^,^49^,^50^,^51^. Considering these severe changes in the plant during chilling acclimation, it is plausible that also during the deacclimation phase at 20 °C many genes need to be transcriptionally reprogrammed, including recovery-specific genes that are involved in specific biochemical pathways mediating, for instance, the metabolism of chilling-specific compounds. Nevertheless, only a low percentage of genes (~5%) that is still differentially regulated during the deacclimation phase shows an overlap with the set of genes that is deregulated in feeding-induced, previously chilled plants. Hence, the differences between the feeding-induced transcriptome of previously chilled and unchilled plants is not simply due to maintained transcriptional changes of chilling-induced genes during the deacclimation phase. Instead, our data suggest that information about the previous chilling is maintained at a level other than the transcriptome. Yet, this information affects the transcriptional responses to feeding damage. Several studies show that plants keep information about a prior cold exposure at the epigenetic, protein and metabolite level for some time^25^,^28^,^52^. Future studies are needed to disentangle by which mechanism the information that is maintained during cold deacclimation affects the feeding-induced transcriptome of *A. thaliana.*

Environmental conditions are constantly changing in natural habitats and thus, both plants and insects are typically exposed to multiple stresses. The responses of plants to a combination of abiotic and biotic stresses may require the expression of other genes than for responses to any single stress alone^53^,^54^ and result in a cross talk of hormone signalling^55^. Indeed, our data show that the feeding-induced transcriptome of previously untreated plants clearly differs from the one of previously chilled plants. Numerous studies investigated plant responses to simultaneously occurring abiotic stress and herbivory. Many of these studies were testing the plant stress hypothesis which predicts that plants under abiotic stress are more suitable as food for herbivores and show reduced defences^56^. However, a meta-analysis by Koricheva *et al*.^57^ found little support for this hypothesis in its general form. To our knowledge, no previous studies have addressed the question how a plant’s transcriptional state induced by an abiotic cue like chilling affects the plant’s transcriptome when responding to a time-delayed biotic stress like herbivory.

It was tempting to hypothesise that the prior chilling experience prepares the plants for a more efficient defence against herbivory, as has also been suggested by Kim *et al*.^58^. Exposure to cold is known to trigger thickening of the cell wall^59^ and to cause changes in cell wall metabolism, among them e.g. increased biosynthesis of phenolic compounds^60^. While thicker leaf cell walls might impair feeding of especially the tiny neonate larvae, the detrimental effects of phenolic compounds on the digestion of leaf tissue have been shown in several studies^61^. However, in spite of the very different transcriptional reactions of previously chilled and naïve plants to feeding damage, *P. brassicae* larval performance did not significantly differ on these types of plants. Hence, our results show that *P. brassicae*, which is specialised on Brassicaceae, can cope with the responses of the host plant *Arabidopsis* to chilling.

In conclusion, we suggest that the fast reset of a plant’s egg-induced transcriptional changes after removal of eggs is a general phenomenon, since fading of a reliably herbivory-indicating cue also implies fading of impending danger by hatching larvae. Maintenance of a transcriptionally (or physiologically) excited state after a past egg deposition may entail some costs^46^, and thus, become inefficient when removal of eggs reduces the risk of herbivory. Future studies are needed to elucidate whether the lack of effects of the chilling-mediated plant transcriptomic changes on the herbivore’s performance are due to an unchanged plant nutritional quality or to the specialisation of the herbivore to its host plant.

## Methods

### Plant material and growth conditions

*Arabidopsis thaliana* Col-0 seeds were sown on a 3:1 mixture of soil (Einheitserde Typ P; Kausek, Mittenwalde, Germany) and vermiculite (Kausek, Mittenwalde, Germany). After three days stratification at 4 °C the plants grew up in growth chambers under short day conditions (10-h/14-h light/dark cycle, 120 μmol m^−2^ sec^−1^ light intensity, 20 °C, 50% relative humidity). For the treatments, we used seven weeks old plants.

### *Pieris brassicae* rearing

*Pieris brassicae* adults were kept in a flight cage (25 × 62 × 62 cm) in a climate chamber under long day conditions (18-h/6-h light/dark cycle, 220 μmol m^−2^ sec^−1^ light intensity, 23 °C, 70% relative humidity). For oviposition, *A. thaliana* Col-0 plants grown under short day conditions were placed in the flight cage for two days. Neonate larvae were transferred into an acrylic glass box (15 × 16 × 42 cm) and kept in a climate chamber under long day conditions (18-h/6-h light/dark cycle, 160 μmol m^−2 ^sec^−1^ light intensity, 21 °C, 70% relative humidity) until used for the plant treatments. They were fed with savoy cabbage (*Brassica oleracea* convar. *capitata* var. *sabauda*) until pupation.

### Plant treatments

To compare the transcriptional responses of *A. thaliana* to insect egg deposition or chilling as primary stimuli followed by herbivory as secondary stimulus, we used the experimental setup depicted in Fig. 1a. We here refer to the primary stimulus as “P” stimulus (PE for egg deposition, P* for chilling) and to the secondary stimulus (always herbivory) as response-triggering “T” stimulus.

For treatment with the primary stimulus “egg deposition”, a mated *P. brassicae* female was prompted to deposit one egg cluster (~40 eggs) on the bottom side of the apex of leaves 16 and 17. The egg-deposited plants were kept for five days under short day conditions (8-h/16-h light/dark cycle, 120 μmol m^−2^ sec^−1^ light intensity, 20 °C, 50% relative humidity). Afterwards the egg clusters were gently removed by peeling them off the leaf. We used wide-tip entomology forceps which were carefully pushed between eggs and leaf without damaging the leaf surface or the eggs.

For treatment with the primary stimulus “chilling”, the seven-week-old plants grown under short day conditions were transferred for five days to 4 °C under short day conditions (8-h/16-h light/dark cycle, 120 μmol m^−2 ^sec^−1^ light intensity, 50% relative humidity). Control plants were kept under the same conditions except for 20 °C ambient temperature. After five days in the cold, plants were retransferred to 20 °C.

Treatment with the secondary triggering stimulus (herbivory) was applied one day after removal of the eggs or one day after retransfer of plants to 20 °C. The removed eggs were transferred to a Petri dish with filter paper and kept under short day conditions until the larvae hatched six days after oviposition. For larval feeding, 20 *P. brassicae* larvae were transferred with a dampish soft brush to leaves 16 and 17 of untreated, chilling-treated or oviposition-treated plants (in total 40 larvae per plant). To prevent escape of the larvae, they were confined to plexiglass clip cages (2 cm *∅*, 1.7 cm high) that covered the leaf area in immediate proximity to the site where previously the egg cluster was located. Hence, larvae had no chance to feed upon leaf tissue where the egg cluster was located before. Larvae which were placed on chilling-treated or untreated plants were also enclosed in clip cages (Fig. 1b). To compensate for potential influences on the leaves by fixation of the clip cages, also the leaves 16 and 17 of untreated, chilling-treated and egg-treated plants not exposed to larvae were caged (Fig. 1b, samples C_2_, C_3_, P_2_*, P_3_*, P_2_E and P_3_E). The larvae were allowed to feed gregariously within the clip cage for two days.

We determined the weight of gregariously feeding *P. brassicae* larvae that had been placed as neonate larvae on leaves (i) from which eggs had been removed one day before exposure to the larvae (Fig. 1a, leaf samples P_3_E + T) or (ii) which had been exposed to chilling followed by deacclimation for one day prior to herbivory (Fig. 1a, leaf samples P_3_* + T). Larval weight was determined after a two-day-feeding period. Furthermore, we determined the plant damage inflicted by the larvae and measured the leaf area consumed by the larvae by taking photographs of the feeding-damaged leaves side by side with a 1 cm^2^ paper square as reference. The number of pixels of the consumed leaf area was approximated by using ImageJ^62^ and converted in mm^2^ by division through the pixel number of the reference.

### Sampling of leaf material and RNA preparation

We sampled leaf tissue for RNA extraction at three time points after treatment. For each time point, we used a different set of plants. After treatment with the primary stimuli “chilling” or “egg deposition” for five days, tissue from the treated leaves was harvested for total RNA extraction (Fig. 1: sampling time point 1; chilling treated plants: P_1_*, egg treated plants: P_1_E, untreated control plants: C_1_). One day after returning to 20 °C or after egg removal, respectively, leaf material was also harvested from a different set of plants (Fig. 1: sampling time point 2; chilling treated plants: P_2_*, egg treated plants: P_2_E, untreated control plants: C_2_). After another two days of treatment with the triggering stimulus “herbivory”, leaf material from herbivore damaged (T) and non-damaged plants was harvested (Fig. 1: sampling time point 3; chilling treated plants: P_3_* and P_3_* + T, egg treated plants: P_3_E and P_3_E + T, untreated control plants: C_3_ and T). Leaf samples were collected by excising a 1 cm wide leaf strip next to the area covered with the clip cage (Fig. 1b). In total, we collected leaf tissue of each treatment at each time point, resulting in 12 types of samples (Fig. 1a). For each type of sample, we harvested tissue of *n* = 3 plants from pooled leaves 16 and 17 and generated 3–4 biological replicates.

Leaf material was frozen in liquid nitrogen and pulverised (3 × 30 sec, maximum frequency) in a ball mill MM 400 (Retsch, Haan, Germany). Total RNA was extracted by using the NucleoSpin^®^ RNA Plant kit (Macherey-Nagel, Düren, Germany) following the manufacturer’s protocol. The purified RNA was subjected to an additional RNase-free DNase I (Thermo Fisher Scientific) digestion step for 30 min at 37 °C to remove any residual DNA. RNA concentration was determined spectrophotometrically, and RNA integrity was verified by gel electrophoresis on denaturing 1.2% agarose-formaldehyde gels.

### cDNA synthesis and quantitative real-time PCR

Total RNA was treated with DNase I (Thermo Scientific) according to the manufacturer’s instructions. First-strand cDNA was synthesised from 2 μg total RNA with SuperscriptTM III Reverse Transcriptase (Invitrogen) and quality controlled following the manufacturer’s instructions. qRT-PCR reactions were conducted on a Stratagene MX3005p Real-Time PCR System (StrataGene Systems, Washington, USA) using the Power SYBR^®^ Green PCR master mix (Applied Biosystems) and following the thermal profile: 1 × (95 °C for 10 min); 40 × (95 °C for 20 s and 60 °C for 60 s). Relative expression levels were calculated according to Livak and Schmittgen^63^ with *AtACT2* (AT3G18780) as reference gene. Primer sequences are listed in Supplementary Table S5.

### Microarray analysis

Expression analysis was performed on ArrayXS Arabidopsis v2 (XS-5010) microarrays in the Agilent 8 × 60K format (Oaklabs GmbH, Hennigsdorf, Germany) that represent 30,541 *Arabidopsis* genes. The array design is described in GEO accession GPL19779. Labelling of total RNA and microarray processing were performed by Oaklabs GmbH. Briefly, the RNA quality was re-assayed on an Agilent 2100 Bioanalyzer. Cy3-labeled cRNA was synthesised with the Agilent Quick Amp Labeling Kit one-color and hybridised to the microarrays according to manufacturer’s instructions. Microarrays were scanned on an Agilent High-Resolution Scanner G2505C, and the images were processed with the Agilent Feature Extraction software using default settings. Expression data were analysed using the Bioconductor Linear Models for Microarray Data (*limma*) software package^64^. For background correction and inter-array normalisation of all 36 array data sets, the “normexp” and “quantile” functions were used, respectively. Features with lower intensity values than ‘maximum dark corner intensity’ were excluded from further analysis.

### Statistical analysis

The data were statistically evaluated by using the software ‘R’^65^. Larval performance parameters were averaged per plant to avoid pseudo-replication and analysed by Mann-Whitney*-U*-tests. Gene expression of the background-corrected and normalised microarray data was assessed according to Smyth^66^. The *P*-values of ≥2-fold up- or downregulated genes were adjusted using Benjamini and Hochberg false discovery rate procedure. Genes with *P*_*adj*_-value < 0.05 were defined as differentially expressed. Identification of significantly enriched gene ontology (GO) terms with *P*_*adj*-_values ≤ 0.05 according to Benjamini and Hochberg false discovery rate calculation was conducted using the DAVID bioinformatics resource tools (http://david.abcc.ncifcrf.gov)^67^.

## Additional Information

**Accession codes:** Microarray gene expression data, metadata and array platform design are deposited in the NCBI Gene Expression Omnibus repository (GEO) under the accession number GSE69623.

**How to cite this article**: Firtzlaff, V. *et al*. Pre-exposure of *Arabidopsis* to the abiotic or biotic environmental stimuli “chilling” or “insect eggs” exhibits different transcriptomic responses to herbivory. *Sci. Rep.* **6**, 28544; doi: 10.1038/srep28544 (2016).

## Supplementary Material

Supplementary Table S1

Supplementary Table S2

Supplementary Table S3

Supplementary Table S4

Supplementary Table S5

## Figures and Tables

**Table 1:** Weight of neonate *Pieris brassicae* larvae and the leaf area consumed by them (means ± SD) on *Arabidopsis thaliana* plants that received egg depositions or a chilling treatment.

Primary stimulus^1^	Secondary triggering stimulus^2^	Treatment code	Larval weight (mg/larva)	*n*^3^	*P*^4^	Consumed leaf area (mm^2^/larva)	*n*^3^	*P*^5^
None	Feeding	T	0.28 ± 0.03	8	n.s.	8.67 ± 2.77	8	n.s.
Eggs	Feeding^6^	P_3_E + T	0.28 ± 0.03	8		8.15 ± 2.51	8	
None	Feeding	T	0.32 ± 0.04	14	n.s.	5.60 ± 1.78	15	n.s.
Cold	Feeding	P_3_* + T	0.34 ± 0.06	18		6.56 ± 1.62	16	

^1^The primary stimulus was lasting 5 days.

^2^The secondary triggering stimulus started one day after removal of the primary stimulus; larvae were allowed to feed for 2 days.

^3^*n* = number of plants treated (performance data of 15–20 larvae were pooled per plant).

^4^*P* > 0.05: not significant (n.s.) Mann-Whitney *U*-Test.

^5^*P* > 0.05: not significant (n.s.) Student’s *t*-test.

^6^Larvae fed in clip cages on tissue next to the former egg cluster.
